# Sentinel node in ovarian cancer: study protocol for a phase 1 study

**DOI:** 10.1186/1745-6215-14-47

**Published:** 2013-02-15

**Authors:** Marjolein Kleppe, Toon Van Gorp, Brigitte FM Slangen, Arnold J Kruse, Boudewijn Brans, Ivo NA Pooters, Koen K Van de Vijver, Roy FPM Kruitwagen

**Affiliations:** 1 Department of Obstetrics and Gynecology, Maastricht University Medical Centre, PO Box 5800 6202 AZ, Maastricht, The Netherlands; 2 GROW, School for Oncology and Developmental Biology, Maastricht, The Netherlands; 3 Department of Nuclear Medicine, Maastricht University Medical Centre, Maastricht, The Netherlands; 4 Department of Pathology, Maastricht University Medical Centre, Maastricht, The Netherlands

**Keywords:** Sentinel node, Ovarian cancer

## Abstract

**Background:**

The concept of sentinel lymph node surgery is to determine whether the cancer has spread to the very first lymph node or sentinel node. If the sentinel node does not contain cancer, then there is a high likelihood that the cancer has not spread to other lymph nodes. The sentinel node technique has been proven to be effective in different types of cancer. In this study we want to determine whether a sentinel node procedure in patients with ovarian cancer is feasible when the tracers are injected into the ovarian ligaments.

**Methods/Design:**

Patients with a high likelihood of having an ovarian malignancy in whom a median laparotomy and a frozen section analysis is planned and patients with endometrial cancer in whom a staging laparotomy is planned will be included.

Before starting the surgical staging procedure, blue dye and radioactive colloid will be injected into the ligamentum ovarii proprium and the ligamentum infundibulo-pelvicum. In the analysis we calculate the percentage of patients in whom it is feasible to identify sentinel nodes. Other study parameters are the anatomical localization of the sentinel node(s) and the incidence of false negative lymph nodes.

**Trial registration:**

Approval number: NL40323.068.12

Name: Medical Ethical Committee Maastricht University Hospital, University of Maastricht

Affiliation: Maastricht University Hospital

Board Chair Name: Medisch Ethische Commissie azM/UM

## Background

Epithelial ovarian cancer (EOC) remains the tumor with the most unfavorable prognosis within the field of the gynecological oncology. The incidence of ovarian cancer in the Netherlands in 2008 was 14.5 per 100,000 with 12.3 deaths per 100,000 [1]. In the United States in 2007 the incidence was 13.0 per 100,000 and there were 8.2 deaths per 100,000 [2]. The high mortality rate is partially due to the fact that approximately 75% of patients are diagnosed with advanced stage EOC.

EOC can metastasize through three different ways: intraperitoneal (in the abdominal cavity), lymphogenous and hematogenous [3,4]. Concerning the lymphogenous spread, it is clear that lymphatic metastases of EOC mainly occur in the para-aortic lymph nodes [5]. It is believed that the tumor cells follow the lymph vessels that accompany the ovarian artery and vein in the infundibulopelvic ligament up to the high para-aortic region and renal vein. Nevertheless, pelvic lymph node metastases are also frequently found [6-16]. These tumor cells probably follow a different route, following the para-uterine vessels in the broad ligament towards the uterine artery and vein and further on to the iliac vessels. In some case reports, isolated inguinal node metastases are also described [17-20]. The exact mechanism of this route of metastasis is still unclear, but the metastatic cells might follow the course of the round ligament towards the inguinal lymph nodes, or might follow the iliac vessels towards the femoral vessels. The incidence of lymph node metastasis in clinical stage I to II ovarian carcinoma is reported between 6.1 - 29.6% (mean 14.2%) [21].

In case of a clinical early stage ovarian cancer, the Dutch guideline [1] recommends a staging laparotomy with adequate lymph node sampling, with an absolute minimum of ten lymph nodes removed. In the same guideline, a footnote is made stating that a larger number of removed lymph nodes will increase the chance of finding metastases. These lymph nodes also need to be sampled from different anatomical regions, of which the most important are the para-aortic and paracaval region between the renal vein and inferior mesenteric artery, the common, internal and external iliac vessels and the obturator fossa.

According to the International Federation of Gynecology and Obstetrics (FIGO), EOC with lymph node metastases is classified as FIGO stage IIIC disease, even in the absence of peritoneal metastases [22]. In contrast to patients with FIGO stage I ovarian cancer after a comprehensive staging procedure, patients with a FIGO stage III ovarian cancer are obliged to receive adjuvant chemotherapy. Therefore, the recognition of lymph node metastases is of utmost importance.

Surgical staging of EOC and the extent of lymph node dissection differs greatly from center to center [20].

A recent review, published in 2011, showed an incidence of 14.2% for lymph node metastasis in early EOC [21]. The incidence is higher in grade 3 tumors (20.0%) and the serous histological subtype (23.3%), whereas in grade 1 and mucinous tumors the incidence is 4.0% and 2.6%, respectively.

The assessment of lymph nodes with the aid of radiological techniques (computed tomography (CT) scan, magnetic resonance imaging (MRI), positron emission tomography (PET)) alone in EOC is insufficient; the sensitivity and specificity for detection of lymph node metastases with PET scan is 73.2% and 96.7%, with CT scan 42.6% and 95.0%, and with MRI 54.7% and 88.3%, respectively [23].

To identify the involved lymph nodes different surgical approaches exist, ranging from taking random lymph nodes in different anatomical regions (lymph node sampling) to a systematic lymphadenectomy [20]. A systematic lymphadenectomy can be considered as the gold standard. However, such a radical procedure gives more morbidity than lymph node sampling. These include the formation of lymphocysts (up to 13.5%), nerve and vessel injury (up to 4%), increased blood loss and increased operating time [24].

With a sentinel node procedure the first node that receives primary lymphatic flow can be identified (the so-called sentinel node). The pathological examination is an indication of the nodal status of the remaining nodes; when the sentinel node is negative, one can presume that the remaining nodes are also not involved. As a consequence, the patient may be spared a radical lymphadenectomy, and thus the associated morbidity [25].

The sentinel node technique has been proven effective in different cancers such as breast cancer and malignant melanoma. In gynecological tumors it has been shown to be effective in vulvar cancer [26]. Currently, sentinel node studies are being performed for cervical and uterine cancer, and some cancer centers already routinely perform sentinel node procedures for these gynecological cancers. Sentinel node studies in ovarian cancer are scarce. Nyberg *et al.* performed a study in 16 patients with high-risk uterine cancer in whom technetium and blue dye were injected into the right or left ovary [27]. Since patients with a high-risk uterine cancer undergo a staging procedure similar to that of patients with early stage ovarian cancer (namely, a total abdominal hysterectomy (TAH) with bilateral salpingo-oophorectomy (BSO) and a pelvic and para-aortic lymphadenectomy), these patients were selected to investigate whether injecting tracers in the ovary would render sentinel nodes. After an incubation time of 15 minutes, a sentinel node was detected in 15 out of 16 patients. Negishi *et al.* used activated charcoal solution to identify ovarian lymphatics in 11 patients [12]. The charcoal was injected into the cortex of the ovary. The charcoal was deposited in (sentinel) lymph nodes of all patients. In both studies the tracer was injected into the ovary. Some authors claim that injecting in the ovary can be difficult when bulky ovarian masses are present. Furthermore, it is claimed that there is a risk of tumor dissemination when tracers are injected into the ovarian capsule [26]. In the current feasibility study, the injection of the tracer is performed in the ovarian ligaments, not in the ovarian cortex. This is to avoid spillage and to be as close as possible to the draining lymph vessels in the ovarian ligaments, irrespective of the size of the ovarian masses. Therefore, in addition to including patients with high-risk endometrial cancer, we also can include patients with an enlarged ovary without risk of tumor dissemination.

Lymphatic mapping can be performed with blue dyes as well as with radioactive isotopes; both can be injected into the ovarian ligaments, which contain the main routes of lymph drainage. After the incubation time the sentinel nodes can be visualized by either colorization (blue lymph nodes can be identified) and/or with a gamma probe that detects the radioactive tracer [27]. In breast cancer it has been shown that the detection rate is highest when both radioactive isotope and blue dye are combined [28,29].

The blue dyes can cause an allergic reaction, exhibited with urticaria, erythema, hypotension and even cardiovascular collapse with bronchospasm [25]. However, the incidence of allergic reactions is very low and varies between 0.07 and 2.7% [30-32].

The radioactive isotope is safe for patient and health care workers [33]. No allergic reactions have been described due to the radioactive isotope.

Since lymph node metastases are found in 14.2% of patients with clinical early stage ovarian cancer, this is a clinically relevant study. Indeed, a sentinel node procedure can prevent unnecessary radical lymph node dissection with the associated morbidity. It could also be more accurate than at-random lymph node sampling which is the current standard care in ovarian cancer in the Netherlands.

This study is scientifically relevant since injecting tracers in the ovarian ligaments can enhance our knowledge on the lymphatic routes and dissemination sites of ovarian cancer, that is, the anatomical locations of the lymph nodes most likely to be involved in ovarian cancer. Injection of tracer in the ovarian ligaments has, to our knowledge, never been published.

## Methods/Design

The primary objective is to determine whether or not a sentinel node procedure in patients with ovarian cancer is feasible by injecting the tracers into the ovarian ligaments instead of into the ovary itself. The secondary objectives are the anatomical location(s) and number of the sentinel node(s): detected peroperatively with blue dye and with a gamma probe, and the detection of residual lymph nodes 24 hours after surgery with scintigraphy.

In this feasibility study we will include both patients who are believed to have a malignant ovarian tumor as well as patients with a high-grade uterine carcinoma. The latter group of patients can also be included because these patients undergo the same surgical procedure: TAH with BSO and a pelvic and para-aortic lymphadenectomy or lymph node sampling.

The study will end when 20 evaluable patients are included, of which at least ten patients have ovarian cancer. The expected inclusion period is estimated to be two years. If the detection rate of the sentinel node is less than 50%, or when fewer than five patients are included in a year, the study will be ended prematurely.

The study will be performed in the Maastricht University Medical Centre. Patients who are believed to have a malignant ovarian tumor planned for exploratory laparotomy will be asked to participate in the study. When a malignancy is confirmed on frozen section, the sentinel nodes will be removed prior to proceeding with a complete staging procedure. Patients with endometrial cancer in whom a staging laparotomy is planned will also be asked to participate.

### Inclusion criteria

1. Patients with a high likelihood of having a malignant ovarian tumor planned for exploratory laparotomy.

2. Patients with high-grade endometrial cancer for whom a staging laparotomy is planned.

3. Age 18 to 85 years.

### Exclusion criteria

1. Previous surgery on one or both ovaries.

2. Previous vascular surgery of the aorta, caval vein, and/or iliac vessels.

3. Previous lymphadenectomy or lymph node sampling in the iliac or para-aortic region.

4. History of a malignant lymphoma.

5. History of a malignant tumor in the abdominal cavity.

6. Previous allergic reaction to blue dye or human albumin.

7. Pregnant or lactating patients.

### Sample size calculation

Between 24 and 28 patients will be included in the study.

The sample size calculation is based on the fact that:

1. With a group of 20 evaluable patients the study group is large enough to give an answer on whether sentinel node(s) can be detected related to the ovarian lymphatic flow when the tracers are injected into the ovarian ligaments. For this purpose, patients with either an ovarian and / or endometrial malignancy and who are planned for a staging laparotomy can and will be included.

2. Patients with a benign result on a frozen section of the suspicious ovary will be excluded from the study because no lymph node(s) will be resected in these patients. These patients will also be documented and described in the report.

3. Based on retrospective data, for 60% of the patients with a suspicious ovary, the frozen section will confirm a ovarian malignancy.

4. To calculate one of the secondary endpoints (incidence of false negative sentinel lymph node) at least 10 patients with ovarian cancer should be part of the 20 evaluable patients.

### Surgical procedure

#### Patients with a high likelihood of having a malignant ovarian tumor

After making the median incision and opening the abdomen, before starting with removal of the enlarged and suspicious adnex, blue dye and the radioactive isotope will be injected into the ligamentum ovarii proprium (median side) and into the ligamentum infundibulo-pelvicum (lateral side), close to the ovary and just below the peritoneum. Therefore, an injection of blue dye (0.5 mL) will be given ventrally and dorsally in the ligamentum ovarii proprium and the ligamentum infundibulo-pelvicum (total amount 2.0 mL). The same will be done with the radioactive isotope, with each injection of 0.5 mL containing a dose of 20-MBq technetium-99-m-labeled albumin nanocolloid (^99m^Tc-nanocolloid or Nanocoll^®^, GE Healthcare, Eindhoven, The Netherlands). A 15-minute interval will be planned after injection. The exact time interval for detection of positive nodes is unclear. Common practice is to wait for 15 minutes; if in this time period the node is not detected, it is considered negative. In daily practice a wait of more than 15 minutes during surgery is not feasible. At 5 and 10 minutes the radioactivity will be measured along the lymphatic tract to document whether or not sentinel node(s) can be identified at a shorter time interval in future studies. A gamma probe measures the radioactivity. The gamma probe will be the same device in all surgical procedures.

After the 15 minutes time interval the ovarian mass will be removed and presented to the pathologist for a frozen section. If the result is benign, no further actions will be performed in these patients. If the result is malignant, the sentinel node(s) will be identified either (once more) by the radioactive tracer and / or visually (blue dye) after opening the retroperitoneal space. After removal of the sentinel node(s) a complete standard staging procedure will be performed including a comprehensive sampling of other lymph nodes at the different locations.

#### Patients with endometrial cancer for whom a staging laparotomy is planned

After making the median incision and opening the abdomen, blue dye and the radioactive isotope will be injected into the ligamentum ovarii proprium (median side) and the ligamentum infundibulo-pelvicum (lateral side) of one of the ovaries, in the same manner as decribed above in patients for whom there is a high suspicion of an ovarian malignancy. The choice of the ovary (left or right side) will alter between left and right by each patient who is included in the study.

A 15-minute interval will be planned after injection. In this time period at 5 and 10 minutes the radioactivity will be measured along the lymphatic tract to document whether or not perhaps sentinel node(s) can be identified at a shorter time-interval in future studies. The radioactivity is measured by a gamma probe. The gamma probe will be the same device in all surgeries.

After the 15 minutes time-interval the surgical staging procedure starts with a TAH and BSO. After approximately 45 minutes the sentinel node(s) will be identified either (once more) by the radioactive tracer and / or visually (blue dye) after opening the retroperitoneal space. This 45 minutes time interval is chosen to mimic the time interval when a frozen section is performed in case of an ovarian tumor. After removal of the sentinel node(s) a complete standard staging procedure will be performed including a comprehensive at random sampling of other lymph nodes at the different locations.

### After surgery

Depending on the mobility of the patient, a scintigram will be performed 24 hours after surgery at the nuclear department. This scintigram is performed to detect any residual radioactive hot spots. If there is any residual radioactivity this may indicate that sentinel nodes have not been identified during surgery and this therefore gives an indication of the reliability of identifying sentinel nodes during surgery.

### Data collection

#### Patient characteristics

The following information will recorded:

1. Age

2. Surgical findings: tumor side, injection site, time between injection and detection, the number of detected sentinel nodes, anatomical location of sentinel nodes, intensity (gamma counts) of radioactive nodes, complications during surgery, side effects during surgery.

3. Histology: results of the frozen section, tumor type (ovarian or endometrial), differentiation grade, histological results of sentinel and non-sentinel nodes.

4. FIGO stage

5. Postoperative scintigram, residual nodes, location of residual nodes.

**Figure 1:**
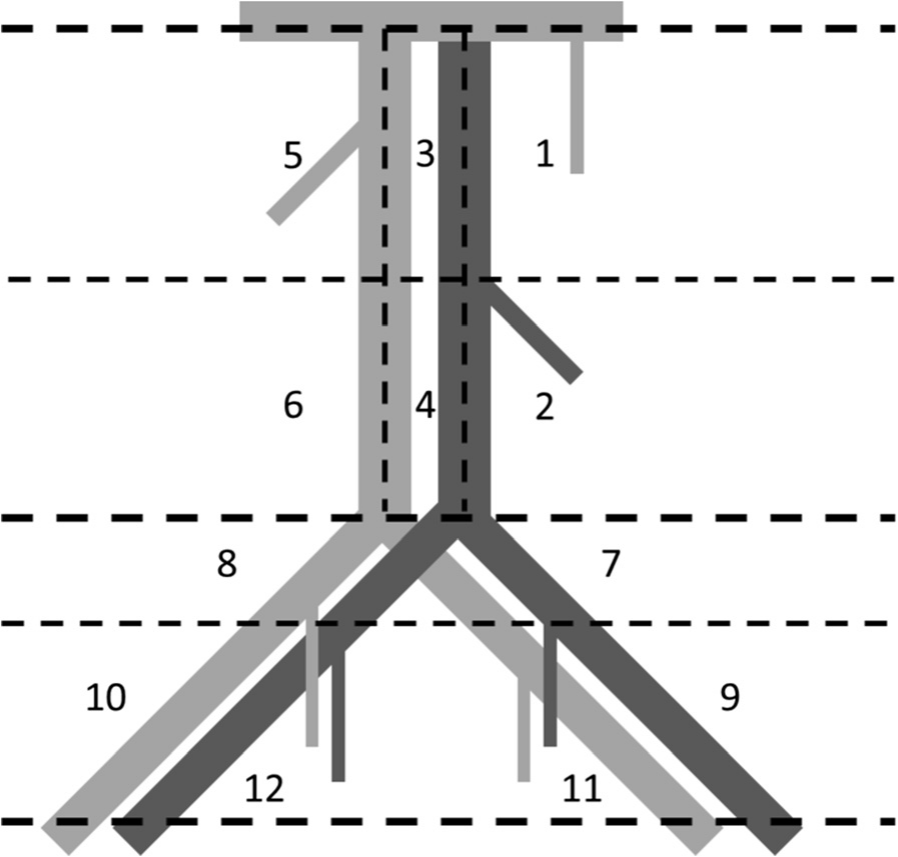
Locations of the sentinel nodes resected.

The surgeon has to register the number and location of sentinel nodes resected. For this purpose the surgeon has to draw the location of the lymph nodes in an anatomical drawing (Figure 1). Twelve different locations will be used to report where the sentinel nodes were found:

1. High para-aortic (left side of the aorta between left renal vein and lower mesenteric artery)

2. Low para-aortic (left side of the aorta below the lower mesenteric artery)

3. High interaortacaval (between aorta and caval vein and between left renal vein and lower mesenteric artery)

4. Low interaortacaval (between aorta and caval vein and below the lower mesenteric artery

5. High para-caval (right side of the caval vein and between right renal vein and above the level of the lower mesenteric artery)

6. Low para-caval (right side of the caval vein and below the level of the lower mesenteric artery)

7. Iliaca communis left

8. Illaca communis right

9. External iliaca left

10. External iliaca right

11. Obturator left

12. Obturator right

The surgeon also has to register the location of the lymphatic tissue removed, related to the lymph node sampling following the removal of the sentinel node(s).

### Withdrawal of individual subjects and replacement

Subjects can leave the study at any time for any reason if they wish to do so without any consequences. The investigator can decide to withdraw a subject from the study for urgent medical reasons. Patients with a benign ovarian tumor on frozen section during surgery will also be excluded from the study. These patients will be replaced by a new study subject until 20 completed sentinel node procedures are performed.

## Discussion

Recognition of sentinel nodes with blue dye and the gamma probe during surgery is less reliable than by making a scintigram. Therefore ideally, as in breast and vulvar cancer, a scintigram is made before surgery to recognize the sentinel nodes so that during surgery no sentinel nodes will be missed. This cannot be accomplished in patients with ovarian cancer, because the tracers are injected during surgery. However, we expect that missing sentinel nodes in patients with ovarian cancer occurs less frequently because the area where the sentinel nodes can occur can easily be visualized. Nevertheless, by making a scintigram 24 hours after surgery, at least an impression is obtained on the incidence of unrecognized sentinel lymph nodes.

### Trial status

Approved by the Medical Ethical Committee Maastricht University Hospital, University of Maastricht. Open for inclusion.

## Abbreviations

BSO: Bilateral salpingo-oophorectomy; CT: Computed tomography; EOC: Epithelial ovarian cancer; FIGO: International federation of gynecology and obstetrics; MBq: Megabecquerel; MRI: Magnetic resonance imaging; PET: Positron emission tomography; TAH: Total abdominal hysterectomy.

## Competing interests

The authors declare that they have no competing interests.

## Authors’ contributions

MK wrote the first version of the manuscript. TVG, AJK, BFMS and RFPMK contributed and revised the manuscript. BB and INAP contributed to the parts of the manuscript concerning nuclear medicine. KKVV contributed to parts of the manuscript concerning the pathology. All authors read and approved the final manuscript.

## Funding

This research received no specific grant from any funding agency in the public, commercial or not-for-profit sectors.
